# 骨转移性LUAD细胞通过HGF/YAP信号通路促进血管生成的机制研究

**DOI:** 10.3779/j.issn.1009-3419.2024.102.38

**Published:** 2024-11-20

**Authors:** Yan DENG, Rong QIU, Xingyu LIU, Yingyang SU, Yang XUE, Yuzhen DU

**Affiliations:** ^1^201306 上海，上海海洋大学水产与生命学院（邓妍，苏英洋，杜玉珍）; ^1^College of Fisheries and Life Science, Shanghai Ocean University, Shanghai 201306, China; ^2^201306 上海，上海交通大学医学院附属第六人民医院医学检验科（邓妍，仇荣，刘星羽，苏英洋，薛阳，杜玉珍）; ^2^Department of Laboratory Medicine, Shanghai Jiao Tong University Affiliated Sixth People’s Hospital, Shanghai 201306, China

**Keywords:** 骨转移肺腺癌细胞, 血管生成, HGF, YAP, Bone-metastatic LUAD cells, Angiogenesis, HGF, YAP

## Abstract

**背景与目的:**

肿瘤骨转移早期与骨微环境血管生态位改变密切相关，异常的血管生成加速肿瘤转移进展，但骨微环境中肺腺癌细胞对血管生态位的作用及其机制仍不清楚。本研究通过探讨骨微环境驯化的肺腺癌细胞株对内皮细胞及血管生成的影响及机制，为骨微环境肿瘤细胞对血管生态位的影响提供参考。

**方法:**

用骨微环境驯化的肺腺癌细胞A549-GFP-LUC-BM3（BM3）培养上清（BM3-CM）和A549-GFP-LUC（A549）培养上清（A549-CM）分别作用于人脐静脉内皮细胞（human umbilical vein endothelial cell, HUVEC），用克隆形成实验、划痕实验及血管生成实验比较HUVEC增殖、迁移及血管生成能力的变化；基因富集分析（gene set enrichment analysis, GSEA）、逆转录定量聚合酶链反应（reverse transcription quantitative polymerase chain reaction, RT-qPCR）以及酶联免疫吸附试验（enzyme linked immunosorbent assay, ELISA）用于检测与细胞血管生成密切相关的肝细胞生长因子（hepatocyte growth factor, HGF）的表达；进一步使用重组蛋白、中和抗体、信号通路抑制剂、免疫荧光染色（immunofluorescence staining, IF）及Western blot等技术验证HGF的关键作用以及分子机制。

**结果:**

BM3-CM比A549-CM具有更强的促HUVEC增殖、迁移及血管生成作用；生物信息学结合体外实验筛选发现分泌型蛋白HGF在BM3细胞及BM-CM中显著增高（P<0.05）；BM3-CM中加入HGF中和抗体可抑制BM3-CM对HUVEC的促进作用（P<0.05），A549-CM中加入HGF重组蛋白可重现BM3-CM对HUVEC的促进作用（P<0.05）；BM3细胞分泌HGF可促进HUVEC的YAP（Yes相关蛋白）活化，该促进作用可能通过激活Src，使YAP入核活化实现（P<0.05）；且促YAP活化作用可被HGF中和抗体所抑制（P<0.05），A549-CM中加入HGF重组蛋白可复现BM3-CM对HUVEC中YAP的活化（P<0.05）。

**结论:**

骨微环境驯化的高骨转移肺腺癌细胞BM3通过HGF/YAP轴促进HUVEC增殖、迁移及血管生成，可能对血管生态位的改变具有重要作用。

## Abstract (en)

**Background and objective:**

The early stages of tumor bone metastasis are closely associated with changes in the vascular niche of the bone microenvironment, and abnormal angiogenesis accelerates tumor metastasis and progression. However, the effects of lung adenocarcinoma (LUAD) cells reprogrammed by the bone microenvironment on the vascular niche within the bone microenvironment and the underlying mechanisms remain unclear. This study investigates the effects and mechanisms of LUAD cells reprogrammed by the bone microenvironment on endothelial cells and angiogenesis, providing insights into the influence of tumor cells on the vascular niche within the bone microenvironment.

**Methods:**

The culture media from bone-metastatic LUAD cell A549-GFP-LUC-BM3 (BM3-CM) and A549-GFP-LUC (A549-CM) were separately applied to human umbilical vein endothelial cell (HUVEC). A colony formation assay, scratch assay, and tube formation assay were conducted to evaluate the proliferation, migration, and angiogenesis of HUVEC. Gene set enrichment analysis (GSEA) was conducted to identify enriched pathways, while reverse transcription quantitative polymerase chain reaction (RT-qPCR) and enzyme linked immunosorbent assay (ELISA) were performed to quantify hepatocyte growth factor (HGF), a protein that plays a crucial role in angiogenesis. Furthermore, the pivotal function of HGF and its underlying molecular mechanisms have been substantiated through the utilisation of recombinant proteins, neutralising antibodies, pathway inhibitors, immunofluorescence staining, and Western blot.

**Results:**

BM3-CM demonstrated a more pronounced impact on the proliferation, migration, and angiogenesis of HUVEC compared to A549-CM. Bioinformatics analysis, combined with in vitro experiment, demonstrated that the secretory protein HGF was significantly elevated in BM3 cells and BM3-CM (P<0.05). The addition of HGF neutralizing antibodies to BM3-CM inhibited the promoting effect of BM3-CM on HUVEC (P<0.05), while the addition of recombinant HGF to A549-CM reproduced that promoting effect of BM3-CM on HUVEC (P<0.05). HGF can enhance the activation of YAP (Yes-associated protein) in HUVEC, and this promotion effect may be achieved by activating Src and activating YAP into the nucleus (P<0.05), but this effect can be inhibited by HGF neutralizing antibodies (P<0.05). Furthermore, the addition of recombinant HGF to A549-CM can recapitulate the YAP activation effect of BM3-CM in HUVEC (P<0.05).

**Conclusion:**

Bone microenvironment reprogrammed bone-metastatic LUAD cells BM3 promote the proliferation, migration, and angiogenesis of HUVEC through the HGF/YAP axis, potentially playing a significant role in the modifications of the vascular niche.

骨是肺腺癌常见的转移部位，超过三分之一的肺腺癌患者会发生骨转移，继发严重的并发症^[1,2]^。骨微环境中血管生态位不仅为血管形成和稳定提供结构基础，还在正常生理及病理过程中发挥重要作用^[3]^。有研究^[4]^表明播散肿瘤细胞优先定殖于富含H型血管的骨干骺端，定殖后的肿瘤细胞与血管生态位互相作用，为转移创造了一个适宜的土壤^[5,6]^。血管生态位对理解肿瘤骨转移进展非常重要，然而，肺腺癌细胞进入骨微环境对血管生态位的影响尚不明确。

本课题组前期用小鼠体内驯化方式获得高骨转移肺腺癌细胞株BM3，其亲骨转移性较亲本肺腺癌细胞株A549显著增强^[7]^。本研究利用体内骨微环境驯化的高骨转移肺腺癌细胞株BM3，以亲本肺腺癌细胞株A549为对照，探讨骨微环境驯化的肺腺癌细胞株对内皮细胞及血管生成的影响及机制，为骨微环境肿瘤细胞对血管生态位的影响提供参考。

## 1 材料和方法

### 1.1 细胞系和主要试剂

本实验所用人肺腺癌细胞系A549-GFP-LUC、人脐静脉内皮细胞（human umbilical vein endothelial cell, HUVEC）购自上海中乔新舟生物科技有限公司，高骨转移肺腺癌细胞株A549-GFP-LUC-BM3由课题组前期分离获得（专利申请号：202311464185.7），DMEM培养基、青霉素-链霉素溶液（P/S）、0.25% Trypsin-EDTA购自美国Gibco公司；胎牛血清（fatal bovine serum, FBS）购自依科赛生物公司；Matrigel基质胶购自美国Corning公司；反转录定量聚合酶链式反应试剂盒（reverse transcription quantitative polymerase chain reaction, RT-qPCR）购自美国EZB公司；BCA蛋白浓度测定试剂盒（增强型）、Western blot及IP蛋白裂解液、PMSF（100 mmol/L）、结晶紫染色液、4’,6-二脒基-2-苯基吲哚（4’,6-diamidino-2 phenylindole, DAPI）、Actin-Tracker Green-488（C2201S, 1:200）购自上海碧云天生物公司；ECL超敏发光检测试剂购自上海雅酶生物医药科技有限公司；Verteporfin购自美国Med Chem Express公司；蛋白Marker（Protein Ladder）购自美国Invitrogen公司；PVDF膜购自Millipore公司；肝细胞生长因子（hepatocyte growth factor, HGF）重组蛋白（#100-39, 30 ng/mL）购自美国Perprotech公司；HGF中和抗体及对照IgG（MAB294, 800 ng/mL）购自美国R&D Systems公司；YAP（#14074T, 1:1000）抗体购自美国Cell Signaling Technology公司；c-Met（25869-1-AP, 1:1000）抗体购自武汉三鹰生物技术有限公司；类固醇受体辅激活因子（steroid receptor coactivator, Src）（WL01570, 1:1000）、p-Src（WL02114, 1:1000）抗体均购自沈阳万类生物科技有限公司；HRP Goat Anti-Rabbit IgG（H+L）（No.AS014, 1:10,000）、Cy3-conjugated Goat anti-Rabbit IgG（H+L）（No.AS007, 1:200）均购自武汉爱博泰克生物科技有限公司；HGF酶联免疫吸附测定法（enzyme-linked immunosorbent assay, ELISA）试剂盒购自南京博研生物科技有限公司。

### 1.2 细胞培养

A549、BM3细胞用含10% FBS的DMEM/F-12（1:1）培养基，HUVEC使用含10% FBS的DMEM培养基，以上细胞均在37 ^o^C、含5% CO_2_的恒温细胞培养箱中培养。

### 1.3 Western blot

收集细胞加入含有PMSF的RIPA裂解液，提取细胞总蛋白，使用BCA法测定蛋白浓度，加入上样缓冲液混匀并在100 ^o^C煮沸10 min。每组取15 μg蛋白在10%的聚丙烯酰胺凝胶中进行电泳，恒流350 mA转PVDF膜，5% BSA室温封闭1 h后，一抗4 ^o^C过夜，孵育后膜用TBST洗涤3次，二抗室温孵育1 h，使用超敏ECL发光液和Western blot 曝光仪器显影，使用Image J软件分析蛋白条带灰度值。

### 1.4 RT-qPCR

使用TRIzol法提取细胞RNA，酶标仪检测其纯度和浓度。按照转录试剂盒说明书进行逆转录和定量。检测mRNA的引物序列见表1。PCR引物由上海生工生物技术有限公司提供。用2^-ΔΔCt^法计算相对表达量。

**表1:** RT-qPCR引物序列

Gene	Sequences (5'-3')
VEGFA	F: AGGGCAGAATCATCACGAAGT R: AGGGTCTCGATTGGATGGCA
VEGFB	F: GAGATGTCCCTGGAAGAACACA R: GAGTGGGATGGGTGATGTCAG
VEGFC	F: GAGGAGCAGTTACGGTCTGTG R: TCCTTTCCTTAGCTGACACTTGT
FGF2	F: AGAAGAGCGACCCTCACATCA R: CGGTTAGCACACACTCCTTTG
PDGFA	F: GCAAGACCAGGACGGTCATTT R: GGCACTTGACACTGCTCGT
PDGFB	F: CTCGATCCGCTCCTTTGATGA R: CGTTGGTGCGGTCTATGAG
ANGPTL1	F: AGTGGACACTGGACATTGCAG R: GCTTCCTCTTTACCATCTGTGG
ANGPTL2	F: GAACCGAGTGCATAAGCAGGA R: GTGACCCGCGAGTTCATGTT
MMP2	F: TACAGGATCATTGGCTACACACC R: GGTCACATCGCTCCAGACT
HGF	F: GCTATCGGGGTAAAGACCTACA R: CGTAGCGTACCTCTGGATTGC

RT-qPCR: reverse transcription quantitative polymerase chain reaction; VEGFA: vascular endothelial growth factor A; VEGFB: vascular endothelial growth factor B; VEGFC: vascular endothelial growth factor C; FGF2: fibroblast growth factor 2; PDGFA: platelet-derived growth factor A; PDGFB: platelet-derived growth factor B;ANGPTL1: angiopoietin like 1; ANGPTL2: angiopoietin like 2; MMP2: matrix metallopeptidase 2; HGF: hepatocyte growth factor.

### 1.5 细胞上清（cell supernatant, CM）收集

取对数生长期的A549细胞和BM3细胞进行消化重悬，以4×10^5^个/皿的密度接种于10 mm培养皿中，待第2天完全贴壁后，使用PBS洗涤3次，加入培养基，培养48 h后收集培养液，2000 g离心15 min后收集上清（A549-CM、BM3-CM）。

### 1.6 Verteporfin（VP）预处理细胞

取对数生长期的HUVEC，消化重悬后以2×10^5^个/孔的密度接种于6孔板，24 h后避光加入YAP抑制剂Verteporfin，浓度为2 mmol/L，以DMSO为对照，锡纸包裹避光培养24 h后进行下一步实验。

### 1.7 克隆形成实验

取对数生长期的HUVEC或Verteporfin预处理后的HUVEC，消化重悬，以1×10^3^个/孔的密度接种于6孔板，24 h后更换条件培养基（对照组、A549-CM组、A549-CM+HGF组、BM3-CM组、BM3-CM+IgG组、BM3-CM+anti-HGF组，上清:完全培养基=1:1），每3天更换新鲜培养基，培养2周后用4%多聚甲醛室温固定30 min，PBS洗涤3次后使用结晶紫染色7 min，PBS再洗涤3次，晾干后于倒置显微镜拍照；使用Image J软件对细胞克隆形成的集落进行计数。

### 1.8 划痕实验

取对数生长期的HUVEC或Verteporfin预处理细胞进行消化重悬，以1×10^5^个/孔的密度接种于6孔板，24 h后更换条件培养基，培养48 h后消化重悬细胞，以2×10^5^个/每孔的密度种于6孔板，第2天当细胞在板底的融合率达到90%时，使用10 μL枪头垂直划痕，用PBS洗涤孔板中漂浮的细胞，分别在0和24 h时在显微镜下拍照。使用Image J软件统计划痕面积。

### 1.9 血管生成实验

4 ^o^C过夜融化基质胶，96孔板中每孔加入50 μL，置于培养箱30 min待其凝固。取对数生长期的HUVEC细胞或Verteporfin预处理细胞进行消化重悬至1×10^5^个/mL浓度，吸取100 μL细胞悬液缓慢加入96孔板内，5 h后在显微镜下拍照。使用Image J进行统一选取各组环形成数和节点进行定量分析。

### 1.10 免疫荧光实验

在24孔板中放入爬片，取对数生长期的HUVEC细胞消化重悬，以3×10^3^个/孔的细胞浓度接种至爬片上，24 h后更换为条件培养基，培养48 h后加入4%多聚甲醛室温固定30 min，0.1% Triton-100破膜10 min，1% BSA封闭30 min，加入YAP一抗4 ^o^C孵育过夜，37 ^o^C避光孵育荧光二抗，PBS洗涤3次后加入Actin-Tracker Green-488室温孵育30 min，洗涤3次，加入DAPI染色5 min。使用荧光倒置显微镜拍照，并在Image J软件上进行相对荧光强度分析。

### 1.11 ELISA

取A549-CM及BM3-CM，根据ELISA试剂盒说明书，在酶标仪检测HGF的含量。

### 1.12 统计学处理

血管生成相关基因集来自MSigDB，富集评分通过基因富集分析（gene set enrichment analysis, GSEA）方法计算。采用Graphpad Prism 9.0对数据进行统计学分析及作图。细胞各实验组每种条件均设置3个独立重复，两组数据之间比较采用t检验，多组数据间比较采用单因素方差分析。P<0.05为差异有统计学意义。

## 2 结果

### 2.1 BM3-CM促进HUVEC增殖、迁移及血管生成

为探究高骨转移肺腺癌细胞BM3对HUVEC增殖、迁移及血管生成的影响，BM3-CM及A549-CM分别作用于HUVEC。平板克隆结果显示，BM3-CM相较A549-CM更能促进HUVEC的克隆形成（P<0.01，图1A、1B）；划痕实验结果显示，BM3-CM可以明显增加HUVEC的迁移能力（P<0.05，图1C、1D）；血管生成实验结果发现，BM3-CM明显促进HUVEC血管形成（P<0.05，图1E、1F）。以上结果表明，BM3-CM具有比A549-CM更强的促HUVEC增殖、迁移及血管生成作用。

**图1:**
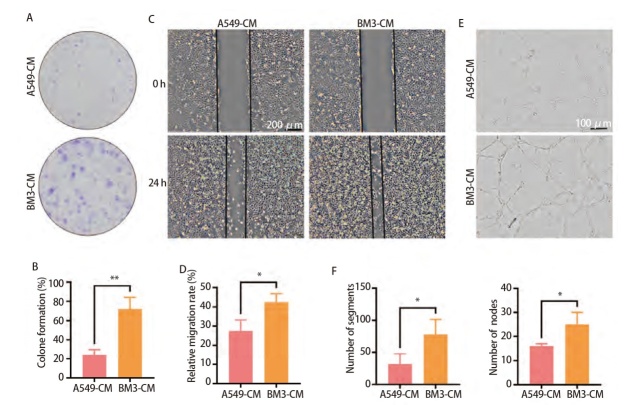
BM3-CM促进HUVEC增殖、迁移及血管生成。A、B：克隆形成实验检测A549-CM和BM3-CM作用HUVEC后HUVEC克隆形成能力；C、D：划痕实验检测A549-CM和BM3-CM作用HUVEC后HUVEC迁移能力（比例尺：200 μm）；E、F：血管形成实验检测A549-CM和BM3-CM作用HUVEC后HUVEC血管生成能力（比例尺：100 μm）。*P<0.05；**P<0.01。

### 2.2 BM3及BM3-CM中高表达HGF

为筛选BM3-CM促进HUVEC细胞血管生成的关键分子，从分子特征数据库（MsigDB）下载血管生成相关基因的基因集，使用GSEA对A549细胞与BM3细胞转录组差异基因进行血管生成相关基因的富集分析，结果显示BM3细胞中血管生成相关基因集上调，并具有统计学意义（P<0.05，图2A）。为了进一步找到与血管生成相关的关键分子，检测A549细胞与BM3细胞转录组中前十血管生成相关基因的表达差异，同时使用RT-qPCR检测相关因子在A549及BM3中的表达量，结果显示，HGF在BM3中表达显著上调（P<0.0001，图2B、2C）。由于HGF为分泌蛋白，使用ELISA对A549-CM及BM3-CM中HGF进行定量，结果显示，HGF在BM3-CM中也显著上调（P<0.001，图2D）。

**图2:**
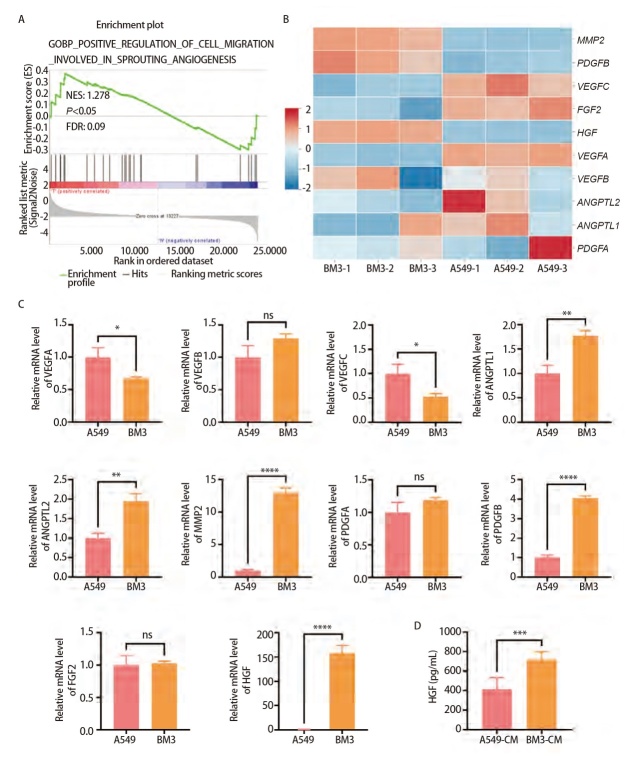
BM3细胞及BM3-CM中高表达HGF。A：A549细胞和BM3细胞的RNA-Seq数据进行的血管生成相关基因富集分析；B：A549细胞和BM3细胞中血管生成相关差异表达基因热图，低水平表达的基因用蓝色表示，高水平表达的基因用红色表示；C：RT-qPCR检测A549细胞和BM3细胞中血管生成相关因子的mRNA 表达；D：ELISA检测A549-CM和BM3-CM中HGF的表达水平。*P<0.05；**P<0.01；***P<0.001；****P<0.0001。

### 2.3 HGF是BM3-CM促进HUVEC增殖、迁移及血管生成的关键分子

为探究HGF是否为BM3-CM促进HUVEC增殖、迁移及血管生成的关键分子，在A549-CM中添加HGF重组蛋白后进行平板克隆实验、划痕实验、血管生成实验，结果显示，HGF重组蛋白能重现BM3-CM的促HUVEC增殖、迁移和血管形成作用（P<0.05，图3A-3F）；进一步，在BM3-CM中加入HGF中和抗体重复上述实验，结果显示，HGF中和抗体使BM3-CM对HUVEC的促进作用被抑制（P<0.05，图3G-3L）。以上实验均说明，HGF是BM3细胞分泌的促进HUVEC增殖、迁移及血管生成的关键分子。

**图3:**
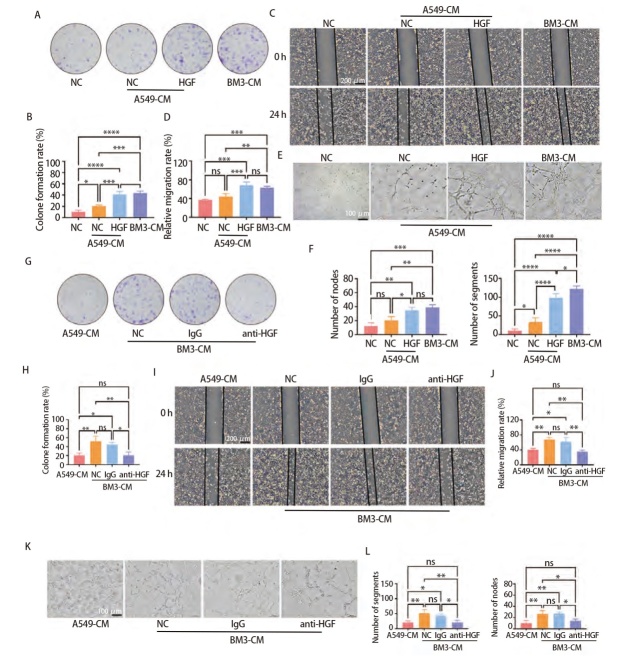
HGF是BM3-CM促进HUVEC增殖、迁移及血管生成的关键分子。A-F：A549-CM中加入HGF重组蛋白对HUVEC增殖、迁移及血管生成的影响，A、B：克隆形成实验，C、D：划痕实验（比例尺：200 μm），E、F：血管形成实验（比例尺：100 μm）；G-L：BM3-CM中加入HGF中和抗体对HUVEC增殖、迁移及血管生成的影响；G、H：克隆形成实验；I、J：划痕实验（比例尺：200 μm）；K、L：血管形成实验（比例尺：100 μm）。*P<0.05；**P<0.01；***P<0.001；****P<0.0001。

### 2.4 BM3通过HGF活化YAP促进HUVEC血管生成

为探究HGF是否通过YAP促进HUVEC血管生成，免疫荧光实验检测经A549-CM和BM3-CM处理的HUVEC核内YAP的表达水平，结果表明，BM3-CM相较A549-CM更能促进HUVEC的YAP入核，使其活化（P<0.001，图4A、4B）。在A549-CM中加入HGF重组蛋白，BM3-CM中加入HGF中和抗体，及使用VP抑制YAP活化后再加入HGF重组蛋白，分别作用于HUVEC，检测HUVEC中YAP、c-Met、p-Src、Src的表达水平，结果表明，HGF可促进YAP活化，该活化作用可能通过激活Src使YAP入核实现（P<0.05，图4C-4F）。同时测定VP抑制YAP活化后，再加入HGF重组蛋白，对HUVEC的增殖、迁移及血管生成能力影响，结果表明，VP抑制YAP活化后，重组HGF不能重现促HUVEC作用（P<0.01，图5A-5F）。以上实验均表明，BM3通过HGF活化HUVEC中YAP，该活化作用可能通过激活Src使YAP入核，进一步促进HUVEC增殖、迁移及血管生成。

**图4:**
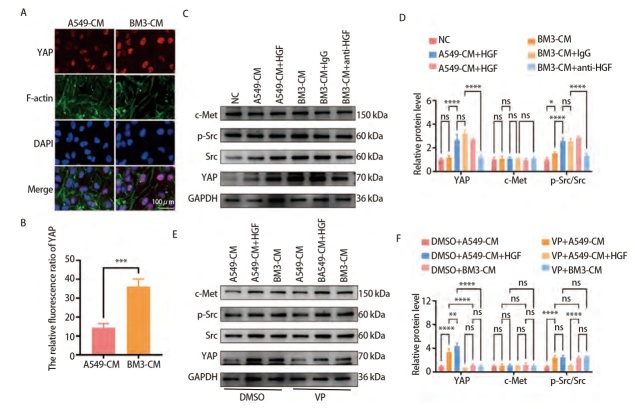
BM3通过HGF激活Src活化YAP。A、B：免疫荧光检测A549-CM和BM3-CM处理HUCEV后HUVEC中YAP表达水平（比例尺：100 μm），YAP（红色）、F-肌动蛋白（绿色）和 DAPI（蓝色）；C、D：Western blot检测不同条件培养基作用HUVEC后YAP、c-Met、p-Src、Src的表达水平；E、F：Western blot检测VP处理HUVEC后再使用不同条件培养基作用HUVEC后YAP、c-Met、p-Src、Src表达水平. *P<0.05；**P<0.01；***P<0.001；****P<0.0001。

**图5:**
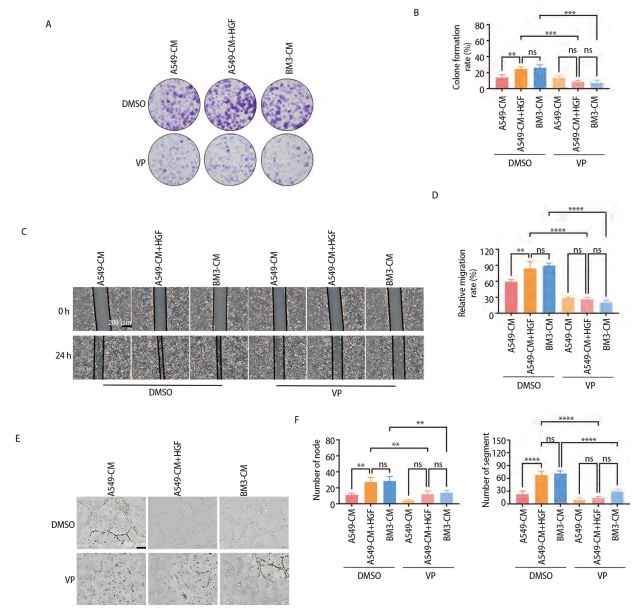
BM3通过HGF/YAP轴促进HUVEC血管生成。A-F：VP处理HUVEC再使用不同条件培养基作用HUVEC后HUVEC的增殖、迁移及血管生成能力；A、B：克隆形成实验；C、D：划痕实验（比例尺：200 μm）；E、F：血管生成实验（比例尺：100 μm）。**P<0.01；***P<0.001；****P<0.0001。

## 3 讨论

血管生态位与肿瘤骨转移密切相关，骨微环境中肿瘤细胞释放刺激骨吸收和血管生成的因子，重塑骨微环境及血管生态位，为肿瘤细胞的转移创造一个更好的土壤，同时骨血管生态位中包含的基质信号选择性地增加骨骼对肿瘤细胞的吸引^[8⇓-10]^。利用骨微环境驯化的肺腺癌细胞探究肿瘤细胞对内皮细胞血管生成的影响，可以为肺腺癌骨转移与血管生态位之间的关系提供参考。本研究结果提示，骨微环境驯化的高骨转移肺腺癌细胞BM3通过HGF/YAP轴促进HUVEC增殖、迁移及血管生成，可能对血管生态位的改变具有重要作用。

本研究所使用的细胞模型为经小鼠内体骨微环境驯化的肺腺癌细胞A549-GFP-LUC-BM3，带有一定的体内表型特性。A549-GFP-LUC通过小鼠左心室注射模拟肿瘤细胞在体内通过血液循环发生骨转移的过程^[11,12]^，从骨转移小鼠模型骨组织分离转移细胞并再注射，经过3次体内驯化最终提取分离获得A549-GFP-LUC-BM3，其体内亲骨转移能力及体外增殖迁移和侵袭能力均显著增强^[7]^。

血管生成过程复杂且受多种因素调控，微环境中肿瘤细胞分泌的生长因子，例如血管内皮生长因子（vascular endothelial growth factor, VEGF）、成纤维细胞生长因子（fibroblast growth factor, FGF）、HGF等都被证实促进肿瘤血管生成^[13]^。在前期研究中，转录组学分析表明HGF是促进血管生成的关键因子，VEGF作为一个重要的促血管生成因子却被发现无显著差异。调研文献^[14]^发现，HGF在干细胞治疗中促血管生成，但VEGF在这一过程中下调，不发挥促血管生成作用，HGF对人真皮微血管内皮细胞的促血管生成作用被发现比VEGF强^[15]^，表明在不同实验体系中，血管生成相关因子的作用是不同的。

HGF过度表达在多种癌症中被发现重塑血管生态位、加速肿瘤发生发展^[16]^。目前研究^[17,18]^发现HGF可通过控制MAPK、PI3K/AKT、β-catenin通路活化血管生成因子、加速血管生成。Hippo信号通路与肿瘤血管生成相关^[19]^，YAP是Hippo通路中关键效应因子，YAP的活化被证明可通过调节血管生成控制骨重塑^[20]^，与骨中血管生态位息息相关。已有文献^[21,22]^表明，HGF可通过受体间质表皮转化因子（c-mesenchymal-epithelial transition factor, c-Met）或Src调控YAP活性。但HGF是否能通过调控YAP促进肿瘤血管生成尚未有过研究，深入研究HGF/YAP对血管生成的作用机制有助于发现骨转移肺腺癌细胞对血管生态位的调控新机制，为肺腺癌骨转移恶性进展的治疗提供可信依据。本研究证实高骨转移肺腺癌细胞培养上清BM3-CM较亲本肺腺癌细胞培养上清A549-CM更能促进血管生成，且高骨转移肺腺癌细胞BM3富集血管生成相关基因，其中HGF是发挥促血管生成的关键因子。同时发现高骨转移肺腺癌细胞培养上清BM3-CM促进HUVEC的YAP活化，该活化作用可能通过激活Src使YAP入核，进而影响HUVEC生物学功能，即高骨转移肺腺癌细胞BM3可通过HGF/YAP轴重塑血管生态位，加速血管生成。

本研究揭示了经过骨微环境驯化的高骨转移肺腺癌细胞通过分泌HGF激活Src活化YAP，促进HUVEC增殖、迁移及血管生成，提示了肺腺癌骨转移时，经骨微环境驯化的肺腺癌细胞可能通过HGF/YAP重塑骨微环境中的血管生态位，形成更多的新生血管支持自身的生长和存活，促进骨转移进展，这为探索肺腺癌骨转移恶性进展分子机制、寻找新的治疗靶点提供了可能。

## References

[1] Siegel RL, Giaquinto AN, Jemal A. Cancer statistics, 2024. CA Cancer J Clin, 2024, 74(1): 12-49. doi: 10.3322/caac.21820 38230766

[2] Zhou CM, Wang ZZ, Zheng Y. Interpretation of US cancer statistics 2023 and its implications for cancer prevention and treatment in China. Zhongguo Aizheng Zazhi, 2023, 33(2): 117-125.

[3] Tang SP, Liao SJ, Huang Q, et al. Current research of the mechanism and influencing factors of type H vessels formation in bone. Zhongguo Jiaoxing Waike Zazhi, 2024, 32(6): 525-529.

[4] Yip RKH, Rimes JS, Capaldo BD, et al. Mammary tumour cells remodel the bone marrow vascular microenvironment to support metastasis. Nat Commun, 2021, 12(1): 6920. doi: 10.1038/s41467-021-26556-6 PMC8626461 34836954

[5] Quail DF, Joyce JA. Microenvironmental regulation of tumor progression and metastasis. Nat Med, 2013, 19(11): 1423-1437. doi: 10.1038/nm.3394 24202395 PMC3954707

[6] Liu ZL, Chen HH, Zheng LL, et al. Angiogenic signaling pathways and anti-angiogenic therapy for cancer. Signal Transduct Target Ther, 2023, 8(1): 198. doi: 10.1038/s41392-023-01460-1 PMC10175505 37169756

[7] Lu Y, Qiu R, Deng Y, et al. Establishment of dual fluorescent labeled human high bone metastasis lung adenocarcinoma cell line and transcriptomic characterization analysis. Zhongguo Feiai Zazhi, 2024, 27(4): 257-265. 38769828 10.3779/j.issn.1009-3419.2024.101.09 PMC11110231

[8] Kusumbe AP, Ramasamy SK, Itkin T, et al. Age-dependent modulation of vascular niches for haematopoietic stem cells. Nature, 2016, 532(7599): 380-384. doi: 10.1038/nature19782 27074508 PMC5035541

[9] Shupp AB, Kolb AD, Mukhopadhyay D, et al. Cancer metastases to bone: Concepts, mechanisms, and interactions with bone osteoblasts. Cancers (Basel), 2018, 10(6): 182. doi: 10.3390/cancers10060182 PMC6025347 29867053

[10] Chen F, Han Y, Kang Y. Bone marrow niches in the regulation of bone metastasis. Br J Cancer, 2021, 124(12): 1912-1920. doi: 10.1038/s41416-021-01329-6 33758331 PMC8184962

[11] Ma L, Sakamoto Y, Kanai A, et al. Characterization of a novel murine colon carcinoma subline with high-metastatic activity established by in vivo selection method. Int J Mol Sci, 2020, 21(8): 2829. doi: 10.3390/ijms21082829 PMC7215277 32325684

[12] Su Y, Luo X, He BC, et al. Establishment and characterization of a new highly metastatic human osteosarcoma cell line. Clin Exp Metastasis, 2009, 26(7): 599-610. doi: 10.1007/s10585-009-9259-6 19363654

[13] Viallard C, Larrivee B. Tumor angiogenesis and vascular normalization: alternative therapeutic targets. Angiogenesis, 2017, 20(4): 409-426. doi: 10.1007/s10456-017-9562-9 28660302

[14] Cheng NC, Tu YK, Lee NH, et al. Influence of human platelet lysate on extracellular matrix deposition and cellular characteristics in adipose-derived stem cell sheets. Front Cell Dev Biol, 2020, 8: 558354. doi: 10.3389/fcell.2020.558354 33195191 PMC7642065

[15] Colin-Pierre C, Berthélémy N, Belloy N, et al. The glypican-1/HGF/c-Met and glypican-1/VEGF/VEGFR2 ternary complexes regulate hair follicle angiogenesis. Front Cell Dev Biol, 2021, 9: 781172. doi: 10.3389/fcell.2021.781172 34957110 PMC8692797

[16] Vimalraj S. A concise review of VEGF, PDGF, FGF, Notch, angiopoietin, and HGF signalling in tumor angiogenesis with a focus on alternative approaches and future directions. Int J Biol Macromol, 2022, 221: 1428-1438. doi: 10.1016/j.ijbiomac.2022.09.129 36122781

[17] Ding X, Xi W, Ji J, et al. HGF derived from cancer-associated fibroblasts promotes vascularization in gastric cancer via PI3K/AKT and ERK1/2 signaling. Oncol Rep, 2018, 40(2): 1185-1195. doi: 10.3892/or.2018.6500 29917165

[18] Chen B, Cai T, Huang C, et al. G6PD-NF-kappaB-HGF signal in gastric cancer-associated mesenchymal stem cells promotes the proliferation and metastasis of gastric cancer cells by upregulating the expression of HK2. Front Oncol, 2021, 11: 648706. doi: 10.3389/fonc.2021.648706 33718248 PMC7952978

[19] Boopathy GTK, Hong W. Role of Hippo pathway-YAP/TAZ signaling in angiogenesis. Front Cell Dev Biol, 2019, 7: 49. doi: 10.3389/fcell.2019.00049 31024911 PMC6468149

[20] Collins JM, Lang A, Parisi C, et al. YAP and TAZ couple osteoblast precursor mobilization to angiogenesis and mechanoregulation in murine bone development. Dev Cell, 2024, 59(2): 211-227. doi: 10.1016/j.devcel.2023.11.029 38141609 PMC10843704

[21] Yan B, Jiang Z, Cheng L, et al. Paracrine HGF/c-MET enhances the stem cell-like potential and glycolysis of pancreatic cancer cells via activation of YAP/HIF-1alpha. Exp Cell Res, 2018, 371(1): 63-71. doi: 10.1016/j.yexcr.2018.07.041 30056064

[22] Farrell J, Kelly C, Rauch J, et al. HGF induces epithelial-to-mesenchymal transition by modulating the mammalian hippo/MST2 and ISG 15 pathways. J Proteome Res, 2014, 13(6): 2874-2886. doi: 10.1021/pr5000285 24766643

